# Supplementary material for: Clinicians’ Perspectives on Barriers and Facilitators for the Adoption of Non-Invasive Liver Tests for NAFLD: A Mixed-Method Study
Source: J Clin Med. 2022 May 11;11(10):2707. doi: 10.3390/jcm11102707 (PMC9146541; doi:10.3390/jcm11102707)
Supplement: Supplementary file 1 [file jcm-11-02707-s001.zip › jcm-1690637-supplementary.pdf]

Supplementary documents

Table S1. Standards for Reporting Qualitative Research (SRQR) checklist

Title and abstract

	Page/line no(s).
**Title** - Concise description of the nature and topic of the study Identifying the study as qualitative or indicating the approach (e.g., ethnography, grounded theory) or data collection methods (e.g., interview, focus group) is recommended	1
**Abstract** - Summary of key elements of the study using the abstract format of the intended publication; typically includes background, purpose, methods, results, and conclusions	3

Introduction

**Problem formulation** - Description and significance of the problem/phenomenon studied; review of relevant theory and empirical work; problem statement	4
**Purpose or research questio**n - Purpose of the study and specific objectives or questions	5

Methods

**Qualitative approach and research paradigm** - Qualitative approach (e.g., ethnography, grounded theory, case study, phenomenology, narrative research) and guiding theory if appropriate; identifying the research paradigm (e.g., postpositivist, constructivist/ interpretivist) is also recommended; rationale**	5
**Researcher characteristics and reflexivity** - Researchers' characteristics that may influence the research, including personal attributes, qualifications/experience, relationship with participants, assumptions, and/or presuppositions; potential or actual interaction between researchers' characteristics and the research questions, approach, methods, results, and/or transferability	5
**Context** - Setting/site and salient contextual factors; rationale**	5, 6
**Sampling strategy** - How and why research participants, documents, or events were selected; criteria for deciding when no further sampling was necessary (e.g., sampling saturation); rationale**	5
**Ethical issues pertaining to human subjects** - Documentation of approval by an appropriate ethics review board and participant consent, or explanation for lack thereof; other confidentiality and data security issues	6

**Data collection methods** - Types of data collected; details of data collection procedures including (as appropriate) start and stop dates of data collection and analysis, iterative process, triangulation of sources/methods, and modification of procedures in response to evolving study findings; rationale**	5,6
**Data collection instruments and technologies** - Description of instruments (e.g., interview guides, questionnaires) and devices (e.g., audio recorders) used for data collection; if/how the instrument(s) changed over the course of the study	5,6
**Units of study** - Number and relevant characteristics of participants, documents, or events included in the study; level of participation (could be reported in results)	7
**Data processing** - Methods for processing data prior to and during analysis, including transcription, data entry, data management and security, verification of data integrity, data coding, and anonymization/de-identification of excerpts	7
**Data analysis** - Process by which inferences, themes, etc., were identified and developed, including the researchers involved in data analysis; usually references a specific paradigm or approach; rationale**	7
**Techniques to enhance trustworthiness** - Techniques to enhance trustworthiness and credibility of data analysis (e.g., member checking, audit trail, triangulation); rationale**	7

Results/findings

**Synthesis and interpretation** - Main findings (e.g., interpretations, inferences, and themes); might include development of a theory or model, or integration with prior research or theory	7-12
**Links to empirical data** - Evidence (e.g., quotes, field notes, text excerpts, photographs) to substantiate analytic findings	7-12

Discussion

**Integration with prior work, implications, transferability, and contribution(s) to the field -** Short summary of main findings; explanation of how findings and conclusions connect to, support, elaborate on, or challenge conclusions of earlier scholarship; discussion of scope of application/generalizability; identification of unique contribution(s) to scholarship in a discipline or field	12,13,14
**Limitations** - Trustworthiness and limitations of findings	13

Other

**Conflicts of interest** - Potential sources of influence or perceived influence on study conduct and conclusions; how these were managed	2
**Funding** - Sources of funding and other support; role of funders in data collection, interpretation, and reporting	2

*The authors created the SRQR by searching the literature to identify guidelines, reporting standards, and critical appraisal criteria for qualitative research; reviewing the reference lists of retrieved sources; and contacting experts to gain feedback. The SRQR aims to improve the transparency of all aspects of qualitative research by providing clear standards for reporting qualitative research.

**The rationale should briefly discuss the justification for choosing that theory, approach, method, or technique rather than other options available, the assumptions and limitations implicit in those choices, and how those choices influence study conclusions and transferability. As appropriate, the rationale for several items might be discussed together.

Table S2. Screening survey

Diffusion and implementation of non-invasive diagnostic liver tests- Screening Survey

Description of the project:

Given the global high prevalence of non-alcoholic fatty liver disease (NAFLD), and due to the limitations of liver biopsy, there is a high interest in developing non-invasive tests to detect NAFLD progression. Multiple non-invasive tests have been developed but actual use in clinics seems to vary substantially.

Different factors play a role when a medical test is introduced into health care practice, and these can influence its dissemination, diffusion, and implementation. In this project we intend to evaluate adopter response rates and diffusion patterns for a number of selected NAFLD tests. We also aim to identify factors that affect test adoption, based on suggestions from industry and practitioners. Using a mixed-methods approach we will evaluate potential explanations for differences in implementation, identifying adaption facilitators and barriers that may slow or block the transition of biomarkers to clinical application as medical tests.

We are currently looking for clinicians from different European countries, who work with NAFLD patients and can help us getting more information on current usage of each test in our list in different clinical settings.

If you are a practitioner working in a clinical setting with NAFLD patients, we would like to invite you to fill in this short screening questionnaire. Based on your availability, we will invite you to participate in a short survey and an online interview.

Background information

Please fill in the following information:

**Name and surname**	
**Age**	
**Country of practice**	
**Name of the hospital/clinic**	
**Specialty**	
**Years of clinical experience**	

Usage of non-invasive liver tests and availability for participation in questionnaire-based survey and an online interview

(1) Are you using any of the tests/scores below for your NAFLD patients in your current clinical practice?

**Test**	**Yes**	**No**
**Fibroscan**	☐	☐
**ELF***	☐	☐
**Fibrotest**	☐	☐
**PRO-C3**	☐	☐
**FIB-4***	☐	☐
**NFS***	☐	☐
**FibroMeter**	☐	☐
**Other – please specify**		

(2) Would you be willing to share your experience and knowledge about these tests by filling in a short questionnaire-based survey and participating in an interview of maximum 30-40 minutes?

	**Yes**
Questionnaire	☐
Interview	☐

*Abbreviations

ELF: Enhanced Liver Fibrosis Test

FIB4: Fibrosis-4 Index for Liver Fibrosis

NFS: Non-Alcoholic Fatty Liver Disease Fibrosis Score

Table S3. Clinicians' responses to the screening survey and the question of whether they use the selected tests for their NAFLD patients in their current clinical practice

	**Total (N=39)**
**FibroScan**	
Yes	39
**FIB4**	
No	5
Yes	34
**NFS**	
No	18
Yes	21
**ELF**	
No	30
Yes	9
**FibroTest**	
No	34
Yes	5
**FibroMeter**	
No	34
Yes	5
**ProC3**	
No	37
Yes	2

Table S4. Topic guide

<table>
<tr><th colspan="3">BARRIERS AND FACILITATORS IN THE IMPLEMENTATION OF NON-INVASIVE NAFLD TESTS
TOPIC GUIDE</th></tr>
<tr><td rowspan="4">Background information</td><td>Interviewer</td><td>Roel Eijk</td></tr>
<tr><td>Supervisor</td><td>Dr. Yasaman Vali (UMC Amsterdam)
Dr. Emmy de Wit (VU University)</td></tr>
<tr><td>Organisation</td><td>UMC Amsterdam – Biomarker Test and Evaluation Program (BiTE)</td></tr>
<tr><td>Period</td><td>01-04-2021 / 01-09-2021</td></tr>
<tr><td>Introduction</td><td colspan="2">• First and foremost, I want to thank you for giving up part of your day to participate in this interview
• I want to provide you with some background information regarding this research project. After that I will explain the way I would like to conduct this interview.
• If you have any questions before starting the interview, you can ask them after this.</td></tr>
<tr><td>Background</td><td colspan="2">• Given the global high prevalence of non-alcoholic fatty liver disease (NAFLD), and due to the limitations of liver biopsy, there is a high interest in developing non-invasive tests to detect NAFLD progression. Multiple non-invasive tests have been developed but actual use in clinics seems to vary substantially</td></tr>
</table>

Goal of this research	• The **goal** of this interview is to better understand the clinician's perspective on barriers and facilitators for the implementation of non-invasive NAFLD tests.<br>• In addition to these interviews, **questionnaires** have been conducted on the barriers and facilitators for implementation of NAFLD tests. Results of both will be compared in order to come to conclusions.
Structure of interview	• The **structure** of this interview will be guided by domains proven important in implementation research, as proposed by the NASSS framework published in 2017 by Greenhalgh et al.
Privacy	• **Data** will be used to (1) be published in a paper and (2) to use in a report for a master thesis.<br>• Your **personal information** will only be seen by our analytical team at UMC Amsterdam.<br>• Furthermore, In our paper, personal information will be **anonymised**. Hence, the results of this research will not be traceable back to you.
Length	• The **length** of this interview will be 30 to 40 minutes.
Questions	• Any further **questions**?
Recording	• This interview will be **recorded** through Zoom. In doing so, I won't have to make notes during the interview and I can listen closely to what you say. It is required by law that I ask your permission to do so. Hence, after starting the recording I will ask for your permission.<br>• START RECORDING<br>• Do I have your **permission** to record this interview?
**INTRODUCTORY QUESTION (5 min.)**	
Background interviewee	• Could you briefly tell me who you are, what your function is and in what sort of clinical setting you work related to NAFLD?
**MAIN QUESTIONS (30 min. – approx. 5 min. per domain)**	
Domain 1:<br>Influence of the Medical Condition	Starting off, factors relating to the medical condition can influence implementation.<br><br>Could you briefly describe the nature of NAFLD as a disease?<br>*Prompts:*<br>• *Is it a complex disease?*<br>• *Are there comorbidities associated with the disease?*<br>• *Are cases often the same, or diverse?*<br>• *Is the disease progression often predictable or unpredictable?*<br>• *Would people with the condition need input from more than one specialist or be in more than one care pathway?*<br><br>How do you think NAFLD and the population it affects might change over the next 3-5 years?<br>*Prompts:*<br>• *Is the prevalence likely to increase or decrease?*<br>• *Is the population affected by NAFLD likely to change?*
Domain 2:<br>Influence of the Technology	Having discussed the disease in question, I would like to talk more about the technology itself as an influence on implementation.

<table>
<tr><td></td><td>Could you describe what kind of information and data the test generates?
Prompts:
How interpretable is the information?
Is the information provided sufficient, and always used in clinical decision making?
Is the information always trusted?
Could you describe what kind of knowledge is needed to use the test?
Prompts:
Is the test itself easy to use?
Is any training required?
To what extent do you think the test implies changes to the way healthcare is delivered?
Prompts:
To what extent does implementation of the test require staff to do their job in a different way?
To what extent does implementation of the test require new or different steps in the care pathway?</td></tr>
<tr><td>Domain 3:
Influence of the Value Proposition (costs and benefits of the innovation)</td><td>The next domain I would like to discuss is the influence of the Value Proposition (i.e. the costs and benefits of the test).

What is known about the value that this test can bring to healthcare practice?
Prompts:
Has this test shown advantage regarding patient management over existing practice (i.e. liver biopsy)?
Are there specific advantages for the patient?
Are any safety concerns related to the test?
What is known about the cost-effectiveness of the test?
Prompts:
Has it been shown cost-effective for the hospital?
Has it been shown cost-effective for the patient?
Has it been shown cost-effective specifically for the country?
Could the test generate a negative value (i.e. costs are larger than benefits) for some stakeholders?
Prompts:
I.e. test not reimbursed for patients/ hospital.
Is this value proposition likely to change in 3-5 years?
Prompts:
Changes in patient management, less safety concerns, studies into cost-effectiveness, change in reimbursement</td></tr>
<tr><td>Domain 4:
Influence of the Intended Adopter</td><td>Of influence on implementation is also the main adopter of the test: clinicians themselves.

What are personal motives for you to implement this test?
Prompts:
How do you as a clinician benefit?
Most important reason for implementation of test?</td></tr>
</table>

<table>
<tr><td></td><td>What could be reasons for you to not implement this test?
Prompts:
Negative sides of the test?
Changing way of practice?
How do you anticipate that clinician’s perception of the test will change over the next 3-5 years?
Prompts:
Do you think clinicians view of the test will change?
Do you think patient acceptance/willingness for involvement in the pathway will change?</td></tr>
<tr><td>Domain 5:
Influence of the Organisation</td><td>Different factors at the organisational level can also play a role in implementation of the test.

Do you think that there is sufficient capacity in the organisation in general for implementation of innovations?
Prompts:
Are internal relations between management and clinicians good?
Would you describe the management structure as flat and egalitarian or top-down and hierarchical?
Has the organisation a good track record of introducing innovations?
To what extent are there slack resources (money and people) available to channel into innovative projects?
Do you think that the organisation is ready for this particular technology?
Prompts:
Who ultimately decides whether a test gets implemented at your organisation?
Are there any key stakeholders who oppose of the implementation?
Is there budget allocated for implementation of the test?
Are there often enough dedicated time and resources available?
Is there extensive evaluation of the effects possible?
How would you rate the amount of work needed to implement the test?
Prompts:
Is the work for introduction, implementation, and evaluation of the test extensive?</td></tr>
<tr><td>Domain 6:
Influence of the Wider Context</td><td>Lastly, there are also factors outside of the organisation which may have an influence on the implementation of the test.

Are there any political factors that may influence the implementation of the test?
Prompts:
What is the reimbursement situation in your country/clinical setting?
Are clinical guidelines for this test present?
Are there any safety/well-being laws which may influence implementation?</td></tr>
</table>

<table>
<tr><td></td><td>What is the regulatory landscape like in your country?
Prompts:
Is the regulatory landscape for development and implementation of these kinds of tests well-defined?
Do you see opportunities to learn from:
Other organisations which have already implemented or are implementing the test?
Other countries?
How may organized networks play a role in the implementation of the test?
Prompts:
Do you think patient organisations will support or oppose the implementation of the test?
Do you think organized networks of clinicians will support or oppose the implementation of the test?
How do you think these political factors may change in the upcoming 3-5 years?
Prompts:
Development of guidelines?
New regulatory framework?
Change of reimbursement situation?</td></tr>
<tr><td colspan="2" align="center">CLOSING QUESTION (3 min.)</td></tr>
<tr><td>Summary</td><td>We’re getting to the end of this interview. As a final question: could you briefly summarize:
The most important barriers for the implementation of this non-invasive NAFLD test in a clinical setting from your perspective?
The most important facilitators for the implementation of this non-invasive NAFLD test in a clinical setting from your perspective?</td></tr>
<tr><td>Final remark</td><td>Anything else you would like to add that we have not discussed yet?</td></tr>
<tr><td colspan="2" align="center">ENDING OF INTERVIEW (2 min.)</td></tr>
<tr><td>Closing</td><td>Then we have come at the end of this interview.</td></tr>
<tr><td>Thanks</td><td>I want to sincerely thank you very much for your time and participation to this interview. I really enjoyed having this conversation with you, and I hope that you experienced it likewise.</td></tr>
<tr><td>Next steps</td><td>This interview will be transcribed ad verbatim.
Thereafter, I will send a summary of approximately one page for a member check. This will give you the possibility to add anything to what has been discussed.
The main results of this interview will be compared to the main results of the other interviews and questionnaires
After analysis, the results will be reported in my master thesis and in a to be published paper.
Would you like to receive a copy of (1) master thesis and (2) receive a notice if a paper is going to be published?
Are there any further questions you have?
STOP RECORDING</td></tr>
</table>

Table S5. Questionnaire

Survey sheet for healthcare professionals

Description of the project

Given the high global prevalence of non-alcoholic fatty liver disease (NAFLD), and due to the limitations of liver biopsy, there is a high interest in developing non-invasive tests to diagnose NAFLD progression. Multiple non-invasive tests have been developed but actual use in clinics seems to vary substantially.

Different factors may play a role when a medical test is introduced into health care practice, and these could influence its dissemination, diffusion, implementation and adoption. Various theoretical models, such as the Non-adoption, Abandonment, Scale-Up, Spread and Sustainability (NASSS) framework by Greenhalgh et al. (2017), have been developed to better understand the antecedents of clinician's intentions to adopt new technologies.

Using a mixed-methods approach we will evaluate potential explanations for differences in adoption, identifying adoption facilitators and barriers in the transition of biomarkers to clinical application as medical tests.

We are currently inviting clinicians from different European countries, who work with NAFLD patients to help us in identifying factors that facilitate or hamper the adoption of non-invasive NAFLD tests in clinical settings. So, if you are a practitioner working in a clinical setting with NAFLD patients, we would like to invite you to fill in this short questionnaire.

Background

According to Greenhalgh et al.'s (2017) NASSS framework, different factors in different domains can influence the adoption of a health technology in clinical setting. As a clinician working closely with non-invasive NAFLD tests, which factors do you think can play a role as a barrier or facilitator in the adoption of FibroScan, ELF and PRO-C3?

Starting off, please indicate how well you would rate your knowledge about the use of FibroScan, ELF and PRO-C3 in clinical practice on a scale of 1 to 5, with 1 being limited and 5 being very good.

**Level of knowledge**	**Limited**<br>*Never used the test and never read literature*	**Below average**	**Average**	**Good**	**Very good**<br>*Read literature and have used / are using*
	1	2	3	4	5
FibroScan	☐	☐	☐	☐	☐
ELF	☐	☐	☐	☐	☐

PRO-C3	☐	☐	☐	☐	☐

Next, please indicate down below to what extent you agree with the following statements regarding different influences on the adoption of respectively FibroScan, ELF and PRO-C3 in a clinical setting.

**Measured item**		**Strongly disagree**	**Disagree**	**Neither agree nor disagree**	**Agree**	**Strongly agree**
		1	2	3	4	5
**Influence of NAFLD as medical condition**						
The complexity of NAFLD as a disease hinders the adoption of the test in clinical setting.	FibroScan	☐	☐	☐	☐	☐
	ELF	☐	☐	☐	☐	☐
	PRO-C3	☐	☐	☐	☐	☐
If NAFLD cases weren't as diverse as they are, adoption of the test in clinical setting would be easier.	FibroScan	☐	☐	☐	☐	☐
	ELF	☐	☐	☐	☐	☐
	PRO-C3	☐	☐	☐	☐	☐

**Measured item**		**Strongly disagree**	**Disagree**	**Neither agree nor disagree**	**Agree**	**Strongly agree**
		1	2	3	4	5
**Influence of technology**						
A lot of training and support is needed to use this test.	FibroScan	☐	☐	☐	☐	☐
	ELF	☐	☐	☐	☐	☐
	PRO-C3	☐	☐	☐	☐	☐
The data generated by this test are not always used for decision making in NAFLD clinical practice.	FibroScan	☐	☐	☐	☐	☐
	ELF	☐	☐	☐	☐	☐
	PRO-C3	☐	☐	☐	☐	☐
The data generated by this test are not always sufficient for decision making in NAFLD clinical practice.	FibroScan	☐	☐	☐	☐	☐
	ELF	☐	☐	☐	☐	☐
	PRO-C3	☐	☐	☐	☐	☐

The data generated by this test are always trusted.	FibroScan	☐	☐	☐	☐	☐
	ELF	☐	☐	☐	☐	☐
	PRO-C3	☐	☐	☐	☐	☐
There is not enough understanding of the use of this test in the pathway of decision making for NAFLD care.	FibroScan	☐	☐	☐	☐	☐
	ELF	☐	☐	☐	☐	☐
	PRO-C3	☐	☐	☐	☐	☐
Overall, this test is easy to use.	FibroScan	☐	☐	☐	☐	☐
	ELF	☐	☐	☐	☐	☐
	PRO-C3	☐	☐	☐	☐	☐

**Measured item**		**Strongly disagree**	**Disagree**	**Neither agree nor disagree**	**Agree**	**Strongly agree**
		1	2	3	4	5
**Influence of value proposition**						
This test is not a cost-effective option for the organization I work at.	FibroScan	☐	☐	☐	☐	☐
	ELF	☐	☐	☐	☐	☐
	PRO-C3	☐	☐	☐	☐	☐
From my perspective, this test is more advantageous regarding patient management over existing NAFLD clinical practice (i.e. liver biopsy).	FibroScan	☐	☐	☐	☐	☐
	ELF	☐	☐	☐	☐	☐
	PRO-C3	☐	☐	☐	☐	☐
This test has an added value for me as a clinician.	FibroScan	☐	☐	☐	☐	☐
	ELF	☐	☐	☐	☐	☐
	PRO-C3	☐	☐	☐	☐	☐
This test has an added value for the patient.	FibroScan	☐	☐	☐	☐	☐
	ELF	☐	☐	☐	☐	☐
	PRO-C3	☐	☐	☐	☐	☐

**Measured item**		**Strongly disagree**	**Disagree**	**Neither agree nor disagree**	**Agree**	**Strongly agree**
		1	2	3	4	5
**Influence of adopter**						
The use of this test changes the usual practice of my work as a clinician for NAFLD care in a positive way.	FibroScan	☐	☐	☐	☐	☐
	ELF	☐	☐	☐	☐	☐
	PRO-C3	☐	☐	☐	☐	☐
Patients are not always willing to cooperate with this test.	FibroScan	☐	☐	☐	☐	☐
	ELF	☐	☐	☐	☐	☐

	PRO-C3	☐	☐	☐	☐	☐

Measured item		Strongly disagree	Disagree	Neither agree nor disagree	Agree	Strongly agree
		1	2	3	4	5
**Influence of organizational setting**						
There is not enough support and advocacy in the organization for the adoption of this test.	FibroScan	☐	☐	☐	☐	☐
	ELF	☐	☐	☐	☐	☐
	PRO-C3	☐	☐	☐	☐	☐
There are enough time and resources in the organization for the adoption of this test.	FibroScan	☐	☐	☐	☐	☐
	ELF	☐	☐	☐	☐	☐
	PRO-C3	☐	☐	☐	☐	☐
There is not enough allocated budget in the organization for adoption of this test.	FibroScan	☐	☐	☐	☐	☐
	ELF	☐	☐	☐	☐	☐
	PRO-C3	☐	☐	☐	☐	☐
There is a shared vision in the organization between management and clinicians regarding adoption of this test.	FibroScan	☐	☐	☐	☐	☐
	ELF	☐	☐	☐	☐	☐
	PRO-C3	☐	☐	☐	☐	☐
Extensive work is needed to properly adopt this test in clinical practice.	FibroScan	☐	☐	☐	☐	☐
	ELF	☐	☐	☐	☐	☐
	PRO-C3	☐	☐	☐	☐	☐

Measured item		Strongly disagree	Disagree	Neither agree nor disagree	Agree	Strongly agree
		1	2	3	4	5
**Influence of wider context**						
The qualification requirements and regulatory landscape for this test are well-defined in my country.	FibroScan	☐	☐	☐	☐	☐
	ELF	☐	☐	☐	☐	☐
	PRO-C3	☐	☐	☐	☐	☐
Currently, there are no rigorous clinical guidelines for use of this test in my country.	FibroScan	☐	☐	☐	☐	☐
	ELF	☐	☐	☐	☐	☐
	PRO-C3	☐	☐	☐	☐	☐
There is enough reimbursement available for this test in my country.	FibroScan	☐	☐	☐	☐	☐
	ELF	☐	☐	☐	☐	☐
	PRO-C3	☐	☐	☐	☐	☐

Measured item		Strongly disagree	Disagree	Neither agree nor disagree	Agree	Strongly agree
		1	2	3	4	5
**Adaptation over time**						
I think that this test has the potential to be adopted at larger scale for NAFLD care in the future.	FibroScan	☐	☐	☐	☐	☐
	ELF	☐	☐	☐	☐	☐
	PRO-C3	☐	☐	☐	☐	☐

Please indicate here what you think are the most important **barriers** for adoption in clinical practice of respectively (1) FibroScan, (2) ELF and (3) PRO-C3.	
1. FibroScan	
2. ELF	
3. PRO-C3	
Please indicate here what you think are the most important **facilitators** for adoption in clinical practice of respectively (1) FibroScan, (2) ELF and (3) PRO-C3.	
1. FibroScan	
2. ELF	
3. PRO-C3	

Table S6. Characteristics of the respondents to the screening survey.

	**Total (N=39)**
**Age (Years)**	
Mean (Range)	45 (30-68)
Missing	2
**Country of practice**	
Belgium	6
UK	6
France	7
Germany	5
Greece	1
Italy	4
Netherlands	9
Sweden	1
**Years of experience (Years)**	
Mean (Range)	17 (3-36)
Missing	1

Table S7. Self-reported level of knowledge of questionnaire respondents from 0 to 5.

	**ELF (N=27)**	**FibroScan (N=27)**	**PRO-C3 (N=27)**
**Level of knowledge**			
Mean	3.3	4.7	2.9
Number of missing	0	0	1

Table S8. The LITMUS Investigators

**The LITMUS Investigators**	
Newcastle University	Quentin M. Anstee<br>Ann K. Daly<br>Olivier Govaere<br>Simon Cockell<br>Dina Tiniakos<br>Pierre Bedossa<br>Fiona Oakley<br>Heather J. Cordell<br>Christopher P. Day<br>Kristy Wonders
AMC Amsterdam	Patrick M. Bossuyt<br>Hadi Zafarmand<br>Yasaman Vali<br>Jenny Lee
Institute of Cardiometabolism And Nutrition	Vlad Ratziu<br>Karine Clément<br>Rafael Patino-Navarrete<br>Raluca Pais
University Medical Center Mainz	Detlef Schuppan<br>Jörn M. Schattenberg
University of Cambridge	Toni Vidal-Puig<br>Michele Vacca<br>Sergio Rodrigues-Cuenca<br>Mike Allison<br>Ioannis Kamzolas<br>Evangelia Petsalaki
Örebro University	Matej Orešič<br>Tuulia Hyötyläinen<br>Aiden McGlinchey
Center for Cooperative Research in Biosciences	Jose M. Mato<br>Óscar Millet
University of Bern	Jean-François Dufour<br>Annalisa Berzigotti

University of Oxford	Michael Pavlides<br>Stephen Harrison<br>Stefan Neubauer<br>Jeremy Cobbold<br>Ferenc Mozes<br>Salma Akhtar
Perspectum	Rajarshi Banerjee<br>Matt Kelly<br>Elizabeth Shumbayawonda<br>Andrea Dennis<br>Charlotte Erpicume<br>Michaela Graham
Servicio Andaluz de Salud, Seville	Manuel Romero-Gómez<br>Emilio Gómez-González<br>Javier Ampuero<br>Javier Castell<br>Rocío Gallego-Durán<br>Isabel Fernández<br>Rocío Montero-Vallejo
Nordic Bioscience	Morten Karsdal<br>Elisabeth Erhardtsen<br>Daniel Rasmussen<br>Diana Julie Leeming<br>Mette Juul Fisker<br>Per Qvist<br>Antonia Sinisi<br>Kishwar Musa
Integrated Biobank of Luxembourg	Estelle Sandt<br>Manuela Tonini
University of Torino	Elisabetta Bugianesi<br>Chiara Rosso<br>Angelo Armandi
Università degli Studi di Firenze	Fabio Marra
Consiglio Nazionale delle Ricerche	Amalia Gastaldelli
Università Politecnica delle Marche	Gianluca Svegliati
University Hospital of Angers	Jérôme Boursier
Antwerp University Hospital	Sven Francque<br>Luisa Vonghia
Linköping University	Mattias Ekstedt<br>Stergios Kechagias
University of Helsinki	Hannele Yki-Jarvinen<br>Kimmu Porthan
UMC Utrecht	Saskia van Mil
National & Kapodistrian University of Athens	George Papatheodoridis

Faculdade de Medicina de Lisboa	Helena Cortez-Pinto
Università degli Studi di Milano	Luca Valenti
Università degli Studi di Palermo	Salvatore Petta
Università Cattolica del Sacro Cuore	Luca Miele
University Hospital Würzburg	Andreas Geier
RWTH Aachen University Hospital	Christian Trautwein
University of Nottingham	Guruprasad P. Aithal
Antaros Medical	Paul Hockings<br>Moritz Schneider
University Hospitals Birmingham NHS Foundation Trust	Philip Newsome
iXscient	David Wenn
University of Lisbon	Cecília Maria Pereira Rodrigues
Genfit	Christian Rosenquist
Intercept Pharma	Aldo Trylesinski
OWL	Pablo Ortiz
EliEli-Lilly	Kevin Duffin<br>James Perfield<br>Yu Chen
Pfizer	Carla Yunis<br>M. Julia Brosnan<br>Theresa Tuthill
Boehringer-Ingelheim	Corinna Schölch<br>Judith Ertle<br>Ramy Younes
Somalogic	Rachel Ostroff<br>Leigh Alexander
Novo Nordisk	Mette Skalshøi Kjær
Ellegaard Göttingen Minipigs	Lars Friis Mikkelsen
Novartis Pharma AG	Maria-Magdalena Balp<br>Clifford Brass<br>Lori Jennings<br>Miljen Martic<br>Jürgen Löffler
AstraZeneca	Sudha Shankar<br>Richard Torstenson
Echosens	Céline Fournier<br>Anne Llorca

Resoundant	Michael Kalutkiewicz<br>Kay Pepin<br>Richard Ehman
Bristol-Myers Squibb	Joel Myers
HistoIndex	Gideon Ho
Allergan	Henrik Landgren
Gilead	Scott D. Patterson<br>Andrew Billin
RTI-HS	Lynda Doward
Takeda Pharmaceuticals International	Paresh Thakker<br>Guido Hanauer
